# Supplementary material for: Downscaling and validating SMAP soil moisture using a machine learning algorithm over the Awash River basin, Ethiopia
Source: PLoS One. 2023 Jan 13;18(1):e0279895. doi: 10.1371/journal.pone.0279895 (PMC9838832; doi:10.1371/journal.pone.0279895)
Supplement: Attachment — Submitted filename: Reviewer Attachment.pdf [file pone.0279895.s004.pdf]

Dear Editor,

Thank you for considering me as a reviewer for this publication in your esteemed journal of PLOS ONE. This manuscript will be ready for publication when these questions and commands are cleared.

Questions and comments:

1- Insufficient period to measure the impact of climate change on Soil Moisture Active.

2- Researchers should examine soil moisture and other influencing climate factors over time.

3- The study of estimating the roughness of the soil surface is not clear and limited!

4- The researchers did not use the Partial Least Squares regression (PLS) in this research; why?

5- What criteria did the researchers follow to determine the Sample Size for this study?

6- The overall quality of figures must be improved: labeling, font size, etc.
