# Supplementary material for: Downscaling and validating SMAP soil moisture using a machine learning algorithm over the Awash River basin, Ethiopia
Source: PLoS One. 2023 Jan 13;18(1):e0279895. doi: 10.1371/journal.pone.0279895 (PMC9838832; doi:10.1371/journal.pone.0279895)
Supplement: Attachment — Submitted filename: Stat review notes.docx [file pone.0279895.s005.docx]

The present work of “Downscaling and validating SMAP soil moisture using a Machine learning algorithm over the Awash River basin, Ethiopia” the authors have presented a decent work of fine-tuning databases as per requirements through statistical procedures, thereby making this research paper purely of statistical background. However, there are ample scopes of improvements as follows-

Please add continuous line numbers to the whole text for easy detection of modifications and review the entire manuscript to an expert English speaker or a Native English speaker.

A. Abstract Section:
1. “Microwave Remote sensing”- Please mention wavelength/s/range.
2. “Coarse spatial resolution”- Mention the resolution
3. We will be grateful for an explanation of how a strong spatial correlation with the original database helps to deliver “More” soil moisture information than the original one?
4. “Cm^3^.Cm^-3^”, is this unit correct? Please explain.
5. Please avoid using abbreviations in the keyword section. Please use full forms then use abbreviations (Like MODIS in introduction section).
6. State the objectives of the study clearly within abstract section as stated at the end of the introduction section.
B. Introduction Section:
1. Please mention active, passive and active-passive satellite separately under a list.
2. Please differentiate soil moisture monitoring with hydrological monitoring... Is this paper for hydrological monitoring?
3. “However, the complex non-linear relationship between coarse-scale soil moisture product and land surface variables constrained the applicability of these downscaling approaches [13].”- Please explain this sentence properly.
4. ……..”algorithm proposed by [14] and later modified by….” This is not ver scientific way of writing references. Please mention the name, year etc properly. Rectify these types of mistakes from the other parts of the manuscript as well.
5. Please clarify the rationale of Objective 1 with the hypothesis of this study. This is neither clear in introduction nor in discussion.
C. Materials and Method:
1. Please clarify soil sampling methods in detail (depth, season, sampling machinery and lat-long of sampling sites)
2. Please mention proper references for “Satellite Data” section.
D. The authors have done quite excellent job in the result section. However, the Discussion section is very poor and doesn’t meet the objectives at all. It seems it has been written in a hurry.

Please relate the facts of results with the scientific references under discussion. Build 3 different segments for 3 different objectives and a common portion summarizing all three objectives.

Construct the conclusion accordingly as well.
