# Supplementary material for: Downscaling and validating SMAP soil moisture using a machine learning algorithm over the Awash River basin, Ethiopia
Source: PLoS One. 2023 Jan 13;18(1):e0279895. doi: 10.1371/journal.pone.0279895 (PMC9838832; doi:10.1371/journal.pone.0279895)
Supplement: Attachment — Submitted filename: Response to Reviewers.docx [file pone.0279895.s006.docx]

**Response to reviewers**

Dear reviewer;

The review of this manuscript, titled "Downscaling and validating SMAP soil moisture using a Machine learning method over the Awash River basin, Ethiopia," was outstanding and completed very quickly. We would like to express our sincere gratitude. We, the writers, appreciate your efforts in making this paper better. Accordingly, we have all of your comments updated, and the updated comments have been underlined in the updated version of the manuscript. The updated manuscript includes the following corrections for each section listed below.

A. **Abstract section**

- Wavelength ranges of microwave remote sensing has been specified
- “Coarse resolution” in this research work was to indicate the 36km x 36km SMAP soil moisture
- The unit cm3 cm3 is corrected. The unit is expressed in **cm^3^ water per cm^3^ of soil**.
- Objective of the research is mentioned in the abstract section

B. **Introduction section**

- Active, passive, and active-passive satellites were inserted in the introduction section.
- All of the comments under this section are well addressed and corrected as of the reviewer comments and highlighted in the revised manuscript.

C. **Material and Method**

- Soil sampling method is corrected as of the comments
- Satellite data type and source is also indicated the table
- Discussion of the research also improved as of each of the research objective

**Response to reviewer 2**

Dear reviewer 2;

This publication, titled "Downscaling and validating SMAP soil moisture using a Machine learning method over the Awash River basin, Ethiopia," has received a great and prompt assessment. We would like to thank you for that. We, the writers, appreciate your efforts in making this paper better. In accordance with this, we have all of your comments updated, and in the updated version of the document, we have underlined the updated remarks. The revised document includes the following justifications and corrections for each section that is listed below.

1. **Insufficient period to measure the impact of climate change on Soil Moisture Active.**

The paper is just providing high quality soil moisture data for hydrological monitoring. It doesn’t tell us the impacts of climate change on soil moisture or other factors. So, ‘Insufficient period to measure the impact of climate change on Soil Moisture Active’ is not as such relevant to this research work.

2. **The study of estimating the roughness of the soil surface is not clear and limited!**

Soil surface roughness is considered as one of the model predictor variables. Based on literatures soil surface roughness and soil moisture are significantly associated. We have tried to make clear the roughness of the soil in the study region. The modification is incorporated in the revised version of the manuscript.

3. **The researchers did not use the Partial Least Squares regression (PLS) in this research; why?**

We used Random Forest (RF) regression model to predict fine resolution soil moisture product over the study region of Awash River basin. The Random Forest (RF) regression has potential in bootstrapping samples. The main reason why we are selecting this model is indicated in the introduction section of the manuscript. There are a lot of scientific evidences that tells us Random Forest regression is better prediction power than any regression models.

4. **What criteria did the researchers follow to determine the Sample Size for this study?**

Purposive sampling was employed to collect soil samples from the field. For this section, we have cleared and highlighted the changes that we have made to the paper in the revised version of the manuscript.

5. **The overall quality of figures must be improved: labeling, font size, etc.**

The overall quality of figures and text are improved as of the recommendation.
